# Gut Microbiome and Metabolome Changes in Mice With Acute Vestibular Deficit

**DOI:** 10.3389/fcimb.2022.821780

**Published:** 2022-04-04

**Authors:** Feitian Li, Yisi Feng, Hongyan Liu, Dedi Kong, Chi-Yao Hsueh, Xunbei Shi, Qianru Wu, Wei Li, Jing Wang, Yibo Zhang, Chunfu Dai

**Affiliations:** ^1^ Department of Otology and Skull Base Surgery, Eye, Ear, Nose, and Throat Hospital, Fudan University, Shanghai, China; ^2^ Key Laboratory of Hearing Medicine, Ministry of Health, Eye, Ear, Nose, and Throat Hospital, Fudan University, Shanghai, China; ^3^ Department of Otolaryngology, The Second Affiliated Hospital, Zhejiang University School of Medicine, Hangzhou, China; ^4^ Department of Head and Neck Surgery, Eye, Ear, Nose, and Throat Hospital, Fudan University, Shanghai, China

**Keywords:** vestibular deficit, anxiety, gut microbiome, gut metabolome, microbiota–gut–brain axis

## Abstract

Vestibular deficit is a very common disorder in clinical practice and is characterized by vertigo, spontaneous nystagmus, and autonomic nervous symptoms, including nausea, vomiting, and sweating. In addition, the comorbidity of vestibular deficit and anxiety has long been an integral component of the medical literature. Previous studies have suggested that the mechanisms underlying this comorbidity involved overlap of vestibular and cerebellar networks. Emerging evidence has shown that the microbiota–gut–brain axis plays a key role in the regulation of affective disorders. Thus, we hypothesized that the gut microbiota may be involved in the comorbidity of vestibular deficit and anxiety. To verify this, we constructed a unilateral labyrinthectomy mouse model to simulate vestibular deficit. Then, 16S rRNA gene sequencing and liquid chromatography–mass spectrometry (LC-MS) were used to analyze the microbiome and metabolome of the cecal samples collected from mice in the unilateral labyrinthectomy, sham surgery, and control groups. Notably, unilateral labyrinthectomy shaped the composition of the mouse gut microbiome, resulting in increased abundance of *Lachnospiraceae NK4A136 group*, *Odoribacter* and *Roseburia* and decreased abundance of *Prevotella* and *Parasutterella* at the genus level. Tax4Fun functional prediction indicated a decrease in tryptophan metabolism in mice in the unilateral labyrinthectomy group. Moreover, functional correlation of changes in gut microbes and metabolites between different groups showed that the oleamide level was negatively correlated with *Odoribacter* abundance (r = -0.89, p = 0.0002). The butyric acid level was positively correlated with *Parasutterella* abundance (r = 0.85, p = 0.0010). The propanoate level was negatively correlated with *Prevotella* abundance (r = -0.81, p = 0.0020). The 20-HETE level was positively correlated with *Parasutterella* abundance (r = 0.84, p = 0.0013). The altered microbes and metabolites were closely related to the pathogenesis of affective disorders. Our results not only offer novel insights into the vestibular deficit comorbid with anxiety but also build an important basis for future research on this etiology.

## Introduction

Vestibular deficit is characterized by vertigo, spontaneous nystagmus, nausea and vomiting. Some patients even present with diarrhea. The impairment of vestibular function occurs in many vertiginous diseases, including Meniere’s disease, vestibular migraine, and vestibular neuritis. It has been reported that about every fifth adult suffers from vertigo or dizziness (Neuhauser, 2016). According to population-based questionnaire studies, the prevalence of vestibular vertigo is approximately 20–30% of adults (Neuhauser, 2007). As the most common complaint in otolaryngological clinical practice, vestibular vertigo remains a substantial health burden that affects millions of people worldwide. In a cross-sectional study that includes 547 patients with vestibular deficit of various entities, the prevalence of anxiety/phobic disorder comorbidities ranges from 17.2% to 32.6% (Lahmann et al., 2015). In addition, we previously demonstrated that vertiginous patients show a higher prevalence of psychiatric disorders, including anxiety and depression, than healthy subjects (Zhai et al., 2016). *In vivo* experiments further confirmed that vestibular deficit elevated anxiety levels in rats (Zhai et al., 2019). Furthermore, it was reported that anxiety, in turn, aggravated the clinical course of vestibular deficit, highlighting the importance of early identification of concurrent anxiety, which could worsen the long-term outcome of patients with vestibular neuritis (Cousins et al., 2017; Bronstein and Dieterich, 2019). Previous studies using retrograde tracing, anterograde tracing, and functional magnetic resonance imaging (fMRI) suggested that vestibular systems show reciprocal connection with a myriad of anxiety-related brain areas, including the dorsal raphe nucleus, locus coeruleus, parabrachial nucleus and hypothalamus (Balaban and Thayer, 2001; Balaban et al., 2011; Yates et al., 2014; Riccelli et al., 2017). Nevertheless, the molecular mechanism underlying the vicious cycle between anxiety and vestibular deficit remains largely unknown. Therefore, studies aimed at better understanding the underlying pathogenic mechanisms of this comorbidity are urgently needed.

The gut microbiome produces numerous bioactive metabolites, which in turn mediate microbial influence on the host body. Thus, the gut microbiome acts as an endocrine organ and regulates multiple pathophysiological processes and even the functions of distal organs *via* metabolism-dependent pathways (Koeth et al., 2013; De Vadder et al., 2014; Tang et al., 2017). Emerging evidence has shown that the enteric microbiome and the brain communicate *via* various routes, forming the microbiota–gut–brain axis (Cryan et al., 2019). Moreover, the gut microbiome has been implicated in many neurological disorders, including anxiety, depression, autism, Parkinson’s disease, and Alzheimer’s disease (Luna and Foster, 2015; Sampson et al., 2016; Sharon et al., 2019; Wang et al., 2019). Research has shown that the gut microbiota modulates psychiatric conditions by producing neurotransmitters, including gamma-aminobutyric acid (GABA), serotonin, melatonin, histamine, acetylcholine, and catecholamines (Banerjee et al., 2021; Morais et al., 2021; Zhou et al., 2022). Besides the bioactive neurotransmitters, gut microbes also produce short-chain fatty acids (SCFAs), such as propionate, butyrate, and acetate, which can pass through the blood–brain barrier and influence central nervous system (CNS) function by regulating numerous host enzymes, such as histone deacetylases (HDACs) (Park and Kim, 2021). Duan et al. (2021) found that gut microbe-derived butyrate reduces microglial activation and increases dendritic spine density, thus ameliorating social deficits and anxiety in high-fat diet (HFD)-fed mice. In addition, depletion of anti-inflammatory bacteria and enrichment of proinflammatory bacteria in the gut microbiota led to excessive IL-6 and corticosterone expression in the blood, followed by reduced brain-derived neurotrophic factor (BDNF) and elevated NF-κB expression in the brain, finally resulting in neuroinflammation, which could be the pathogenic basis of anxiety and depression (Kim et al., 2020; Nikolova et al., 2021).

Previous studies on vestibular deficit comorbid with anxiety have mainly focused on the neuroanatomical connection of vestibular nuclei and other brain regions. Since the microbiota–gut–brain axis is involved in the regulation of the brain activity, we hypothesized that it may participate in the modulation of the comorbidity of vestibular deficit and anxiety. However, whether vestibular dysfunction elicits gut microbiome alteration is poorly understood and should be investigated. This is a pilot study in which we used a multiomic approach for the first time to investigate the effect of vestibular deficit on the gut microbiome and metabolic profiles in a unilateral labyrinthectomy mouse model.

## Methods

### Animals

Six-week-old male C57/BL6J mice were purchased from Shanghai JSJ Laboratory Animal Co., Ltd. The mice were kept at 21–23°C under a 12-h regular light/dark cycle. All animal procedures were performed in accordance with the experimental protocol approved by the animal ethics committee of the medical school at Fudan University in China.

### Experiment Protocol

Animals were allowed to acclimatize for a week and randomly divided into 3 groups: the control group (CON: n = 5), unilateral labyrinthectomy group (UL: n = 6), and sham surgery group (SS: n = 5). Mice in the UL group underwent surgical intervention with a unilateral labyrinthectomy procedure described in the literature. The main steps of the procedure were as follows: under general anesthesia, a posterior incision of the skin following the external auditory canal anteriorly exposed the facial nerve, external auditory canal, and sternomastoid muscle, which were then protected. The sternomastoid muscle was retracted to expose the mastoid bone. After the mastoid bone was drilled, the posterior semicircular canal became visible and was opened, and the bony defect was enlarged to expose the vestibular cavity. Last, the vestibular cavity was packed with gelatin foam, and the skin was sutured (for details, see Simon et al., 2020). Mice in the sham surgery group underwent the same procedure as those in the UL group except for the destruction of vestibular end-organs. Cecal samples were freshly collected and stored at -80°C until further analysis. Samples of the UL group were collected one week postoperatively. Samples of the SS and CON groups were collected simultaneously as those of the UL group.

### Behavior Evaluation

To assess the impairment of vestibular function, mouse behavior was evaluated before surgery and one day and one week after surgery. The behavior of the mice was scored according to the method described by Simon et al. (2020). A total of 8 items were recorded, namely normal locomotor mouse behavior, including the ability to swim, to groom, to move in the cage, and to reach for food or water; vestibular postural and locomotor impaired behavior, including head tilt, tumbling, twirl, and circling. Head tilt was defined as the mouse inclining its head toward the lesioned side. Tumbling was defined as the mouse rolling around its longitudinal axis toward the lesioned side. Twirl was defined as rotation of the whole body while the mouse was being held by the tail. Circling was defined as a mouse circling around its hips in stereotypical movement. Each item was rated from 0 (normal) to 3 (highest deficit), with a maximum score of 24.

### 16S rRNA Gene Sequencing of the Gut Microbiome

DNA was extracted from stool samples using the cetyltrimethylammonium bromide or sodium dodecyl sulfonate (CTAB/SDS) method. First, the DNA concentration and purity were analyzed on agarose gels. Then, the DNA was diluted to 1 ng/ul with sterile water. Specific primers with the barcode (515F: GTGCCAGCMGCCGCGGTAA and 806R:GGACTACHVGGGTWTCTAAT) and Phusion^®^ High-Fidelity PCR Master Mix with GC Buffer (New England Biolabs, USA) were used to amplify the V4 region of the 16S rRNA gene on a PCR system. The mixture PCR product was purified with a Qiagen Gel Extraction Kit (Qiagen, Germany).

The TruSeq^®^ DNA PCR-Free Sample Preparation Kit (Illumina, USA) was used to generate sequencing libraries according to the manufacturer’s instructions. The library quality was evaluated both on a Qubit^®^ 2.0 Fluorometer (Thermo Fisher, USA) and by Q-PCR. Finally, the library was sequenced on the NovaSeq6000 system (Illumina, USA).

The UPARSE algorithm was applied to analyze the sequences (UPARSE v7.0.1001, http://www.drive5.com/uparse/). Sequences with ≥97% similarity were assigned to the same operational taxonomic units (OTUs). The abundance information of the OTUs was normalized using a standard of sequence number corresponding to the sample with the least number of sequences. Furthermore, alpha and beta diversities were calculated on QIIME (Version 1.9.1). Alpha diversity was used to analyze the complexity of species diversity within a sample through Simpson’s index of diversity, and the Shannon’s index, Chao1 and ACE indexes. Simpson’s index of diversity in this study was calculated as 1-Simpson’s index. Beta diversity was used to evaluate differences in species complexity between samples. Nonmetric multidimensional scaling (NMDS) analysis and hierarchical clustering analysis using the unweighted pair-group method with arithmetic mean (UPGMA) were adopted to identify differences between groups. LEfSe (Segata et al., 2011) was the algorithm used to identify high-dimension bacterial markers from the UL, CON, and SS groups. Subsequently, the predicted functional composition profiles of 16S rRNA sequences were collapsed into Kyoto Encyclopedia of Genes and Genomes (KEGG) pathways using Tax4Fun (Aßhauer et al., 2015).

### Gut Metabolomics Analysis

Liquid chromatography–mass spectrometry (LC-MS) was performed to analyze gut metabolites. The original data file obtained by LC-MS analysis was converted to mzML format by ProteoWizard software. Peak extraction, alignment and retention time correction were performed by the XCMS program. The “SVR” method was used to correct the peak area. Metabolic identification information was obtained by searching the laboratory’s self-built database and integrating the results with information from public database and metDNA. Finally, statistical analysis was carried out by the R program. The statistical analysis includes univariate analysis and multivariate analysis. The univariate statistical analysis includes Student’s t-test and variance analysis for multiple comparisons. The multivariate statistical analysis includes principal component analysis (PCA) and orthogonal partial least-squares discriminant analysis (OPLS-DA). Differential metabolites were screened and the criteria were as follows: variable importance in projection (VIP) scores ≥ 1; *p-value ˂* 0.05; fold change ≥ 2 or ≤ 0.5. Volcano plots, linear discriminate analysis (LDA) effect size charts, and heatmaps were used to illustrate the differences in metabolites between groups. The KEGG database was used to perform pathway enrichment analysis of metabolites in difference.

### Statistical Analysis

All data are presented as mean ± standard deviation (SD). Statistical comparisons between multiple treatment groups were performed using Wilcoxon’s test or Student’s t-test (R software). Spearman correlation tests were applied to identify the correlation between gut microbiome and metabolome. The graphs were constructed by R software (Version 3.5.0) and GraphPad Prism 8. Results with a *p-value ˂* 0.05 were considered to be statistically significant.

## Results

### Establishment of the Unilateral Labyrinthectomy Mouse Model

The behavior of the CON (n = 5), SS (n = 5), and UL (n = 6) groups was scored preoperatively, 1 day, and 7 days postoperatively to demonstrate successful lesion of the vestibule. The behavior signing vestibular impairment was absent in CON and SS groups before and after surgery. In the mice of the UL group, however, this behavior was apparent 1 day postoperation, and the mice gradually recovered by 7 days postoperation (**Table 1**). Therefore, the mouse model of unilateral labyrinthectomy was successfully established.

**Table 1:** Behavioral score.

Lesion	CON	SS	UL
Behavioral score			
Before	0	0	0
Day 1	0	0	21.0 ± 3.5
Day 7	0	0	11.2 ± 2.9

According to Simon et al., a total of eight behaviors were scored from 0 (normal) to 3 (highest deficit): ability to swim, to groom, to move in the cage, to reach for food or water, head tilt, tumbling, twirl, and circling. All values are represented as mean ± SD (standard deviation). CON, control group; UL, unilateral labyrinthectomy group; SS, sham surgery group.

### Unilateral Labyrinthectomy Affected the Community Diversity But Not the Richness of the Gut Microbiome

**Figure 1:**
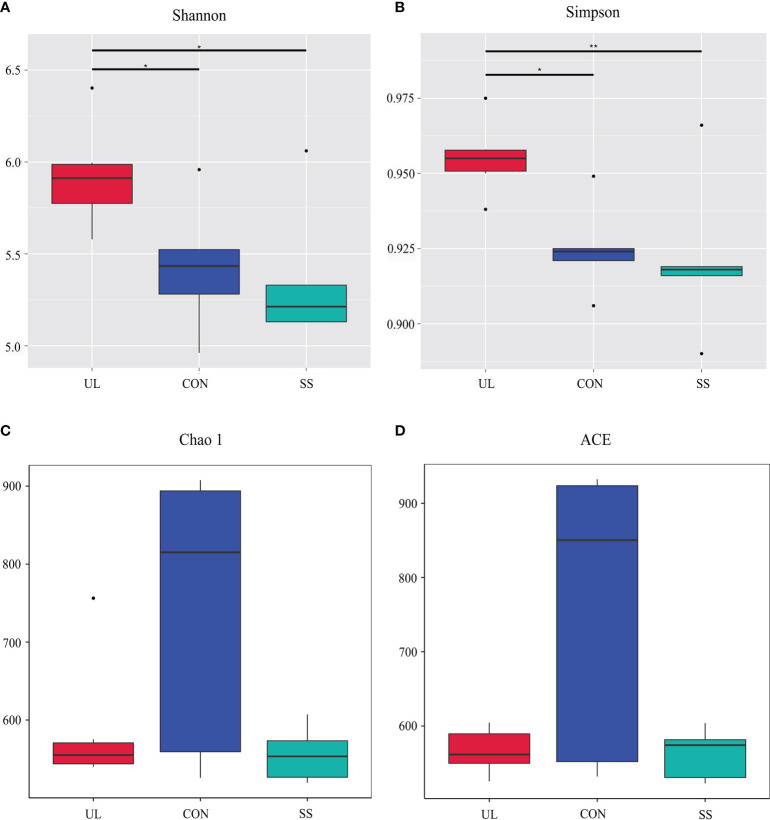
Alpha diversity of gut bacteria. Estimation of community diversity **(A, B)** and richness **(C, D)** is illustrated using boxplots for UL (red), CON (blue), and SS (green) groups. The boxplots show median, 25^th^ and 75^th^ quartile, smallest, and largest values. UL group (n = 6), CON group (n = 5), SS group (n = 5). The Wilcoxon rank-sum test was used to analyze the statistical significance of alpha diversity; *p-value < 0.05 and **p-value < 0.01, versus the CON group. CON, control group; UL, unilateral labyrinthectomy group; SS, sham surgery group.

16S rRNA gene sequencing was used to evaluate the alterations in the gut microbiome induced by unilateral labyrinthectomy. Community diversity and richness were measured by the alpha diversity metrics. The Shannon’s index and Simpson’s index of diversity revealed that the UL group exhibited significantly increased gut microbiome diversity compared to the SS and CON groups (**Figures 1A, B**). However, the richness estimator Chao1 and ACE showed no significant difference among the CON, SS, and UL groups (**Figures 1C, D**).

### Unilateral Labyrinthectomy Shaped the Gut Microbiome Composition

**Figure 2:**
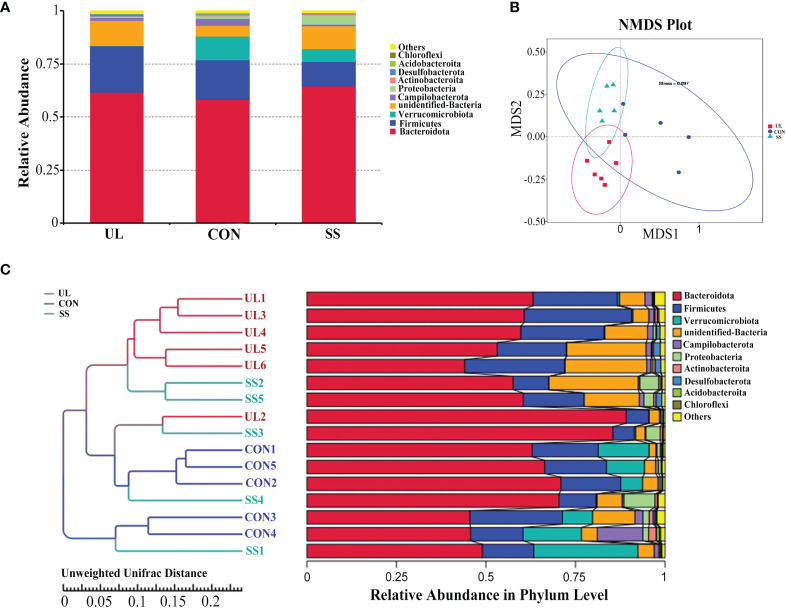
Unilateral labyrinthectomy changes the composition of the gut microbiome. (UL: n = 6, CON: n = 5, SS: n = 5). Composition and relative abundance of bacterial phyla in the three groups **(A)**. Beta diversity was measured by NMDS analysis. The groups showed significant differences in similarity tested by ANOSIM (UL versus SS: R = 0.4293, *p-value* = 0.004; CON versus UL: R = 0.9520, *p-value* = 0.002; CON versus SS: R = 0.4280, *p-value* = 0.007) **(B)**. Composition of each sample at the phylum level with hierarchical clustering based on Unweighted Unifrac Distance of OTU profiles **(C)**. CON, control group; UL, unilateral labyrinthectomy group; SS, sham surgery group.

A total of 1,698 OTUs were detected in the publicly available sequences from the Silva 138 database. From these, 1,695 (99.82%) OTUs were annotated in the OTU database, while 3 (0.18%) were not. The proportions of annotated OTUs at the kingdom, phylum, class, order, family, genus, and species levels were 99.82%, 91.93%, 91.34%, 87.28%, 73.62%, 36.40%, and 6.42%, respectively. *Bacteroidota* was the predominant phylum in the mouse’s gut microbiome, followed by phyla *Firmicutes* and *Verrucomicrobiota*. However, the relative abundance of *Verrucomicrobiota* and *Proteobacteria* was much lower in UL group compared to those in CON and SS groups (**Figure 2A**).

As shown in **Figure 2B**, the 3 groups clustered separately based on analysis of similarities (ANOSIM method) (UL versus SS: R = 0.4293, *p-value* = 0.004; CON versus UL: R = 0.9520, *p-value* = 0.002; CON versus SS: R = 0.4280, *p-value* = 0.007). Furthermore, hierarchical clustering analysis using UPGMA showed that most of the UL, CON, and SS samples were clustered in their own groups (**Figure 2C**).

**Figure 3:**
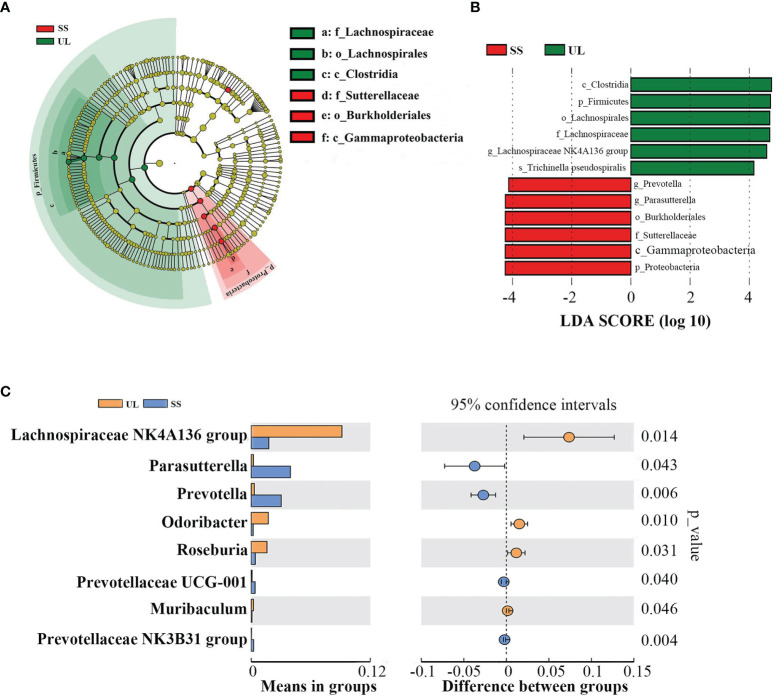
Microbial community dynamics of the gut after unilateral labyrinthectomy. Cladogram representation of the gut microbiota in the UL versus the SS group by 16S rRNA sequencing (UL: n = 6, SS: n = 5). Enriched taxa in the UL (green) and SS (red) groups are indicated. The brightness of each dot is correlated with its LDA effect size **(A)**. LDA coupled with effect size measurements in the UL and SS groups. Enriched taxa in the UL (green) and SS (red) groups are displayed with LDA scores. Only taxa with LDA scores beyond the threshold of 4.0 are shown **(B)**. Statistical analysis of the gut microbiota in UL and SS groups by Student’s t-test **(C)**. UL, unilateral labyrinthectomy group; SS, sham surgery group.

By comparing the gut microbial community between the UL and SS groups using LEfSe analysis, we found that the UL group had a higher abundance of the *Lachnospiraceae NK4A136 group* than the SS group at the genus level. In contrast, the UL group had a lower *Prevotella* and *Parasutterella* abundance than the SS group (LDA score > 4) (**Figure 3A, B**). Moreover, the abundance of *Lachnospiraceae NK4A136* group, *Prevotella, Parasutterella, Odoribacter* and *Roseburia* was also significantly different between the two groups, as determined by Student’s t-test (*p-value* < 0.05) (**Figure 3C**). Meanwhile, the CON group had a higher abundance of *Alloprevotella* and *Lactobacillus* than the SS group (**Supplementary Figure S1**).

### Functional Prediction of the Predominant Taxa

Tax4Fun is an open-source R package designed to estimate the functional capabilities of microbial communities identified in 16S rRNA sequencing, and it allowed functional annotation of the gut microbiome in the datasets of the CON, SS, and UL groups. Tax4Fun function prediction connects the composition with the function of the microbiome. Eight metabolic pathways, which were level 3 KEGG pathways, were differentially enriched among the groups. The gut microbiome of the UL group had a lower abundance of valine, leucine, and isoleucine degradation, tryptophan metabolism, and a higher abundance of NOD-like receptor signaling pathway than the SS group. The CON group had a higher abundance of ribosome biogenesis than the SS group, as determined by Student’s t test (*p-value* < 0.05) (**Supplementary Figure S2**).

### Unilateral Labyrinthectomy Altered the Gut Metabolome

**Figure 4:**
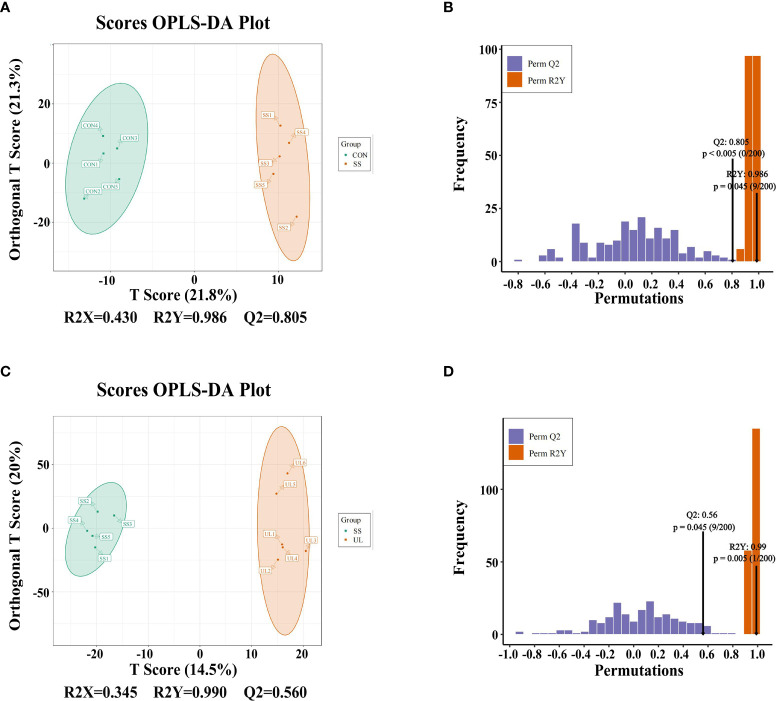
OPLS-DA analysis was performed to show the metabolite profile (UL: n = 6, CON: n = 5, SS: n = 5). OPLS-DA score plot and OPLS-DA model test chart showed good discrimination between CON and SS groups with R^2^X = 0.430, R^2^Y = 0.986, Q^2^ = 0.805, *p-value* < 0.05 **(A, B)**. OPLS-DA score plot and OPLS-DA model test chart showed good discrimination between UL and SS groups with R^2^X = 0.345, R^2^Y = 0.990,Q^2^ = 0.560, *p-value* < 0.05 **(C, D)**. CON, control group; UL, unilateral labyrinthectomy group; SS, sham surgery group.

The gut metabolomic profiles were determined by LC-MS. As shown in **Supplementary Figures S3A–C**, the PCA plot revealed that the metabolites of the CON group were separated from those of the SS and UL groups. An OPLS-DA model was adopted to further analyze the differential metabolites between different groups. The OPLS-DA scores indicated that the UL, SS, and CON groups were separated into different regions (**Figures 4A, C**). Goodness-of-fit values and predictive ability values (CON versus SS group: R2X = 0.430, R2Y = 0.986, Q2 = 0.805, *p-value* < 0.05; UL versus SS group: R2X = 0.345, R2Y = 0.990, Q2 = 0.560, *p-value* < 0.05) indicated that the OPLS-DA model possessed a satisfactory fit with effective predictive power (**Figures 4B, D**).

**Figure 5:**
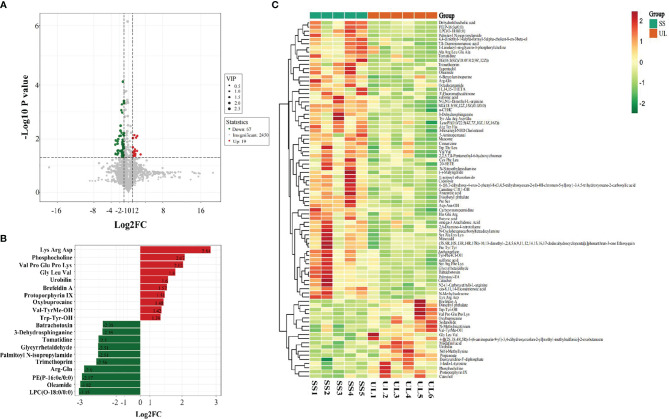
Volcano plot showing differential metabolites between the SS and UL groups **(A)**. Linear discriminant analysis (LDA) effect size (LEfSe) results showed the top 20 differential metabolites between the SS and UL groups **(B)**. Hierarchical cluster analysis of differential metabolites between the SS and UL groups **(C)**. Each column represents a sample, and each row stands for a metabolite. The differential metabolites were identified using the following criteria: variable importance in projection (VIP) ≥ 1.0, *p-value* < 0.05 and fold change ≥ 2 or ≤ 0.5. SS: n = 5, UL: n = 6. UL, unilateral labyrinthectomy group; SS, sham surgery group.

Based on the results of OPLS-DA, our study used the VIP as a threshold to further screen differential metabolites between groups. Metabolites with VIP≥1.0, *p-value* < 0.05, and fold change ≥ 2 or ≤ 0.5 were defined as differential metabolites. Sixty-six differential metabolites were detected in the CON versus SS group, and 86 were detected in the SS versus UL group (**Figure 5**). The levels of 25 metabolites increased significantly, while those of 41 metabolites decreased significantly, in the SS group compared to the CON group. In the UL group, the levels of 19 metabolites increased significantly while those of 67 decreased significantly compared to the SS group (**Supplementary Figure S4**).

To identify the involved metabolic pathways, the differential metabolites were examined against the KEGG database. The results showed that the differential metabolites participated in metabolic pathways involving porphyrin and chlorophyll metabolism, thyroid hormone synthesis, vascular smooth muscle contraction, and choline metabolism in cancer, suggesting that the disturbance of many pathways was implicated in the progression of the unilateral vestibular deficit (**Supplementary Figure S5**).

### Correlations Between the Gut Microbiota and Metabolites in the Unilateral Labyrinthectomy Mouse Model

**Figure 6:**
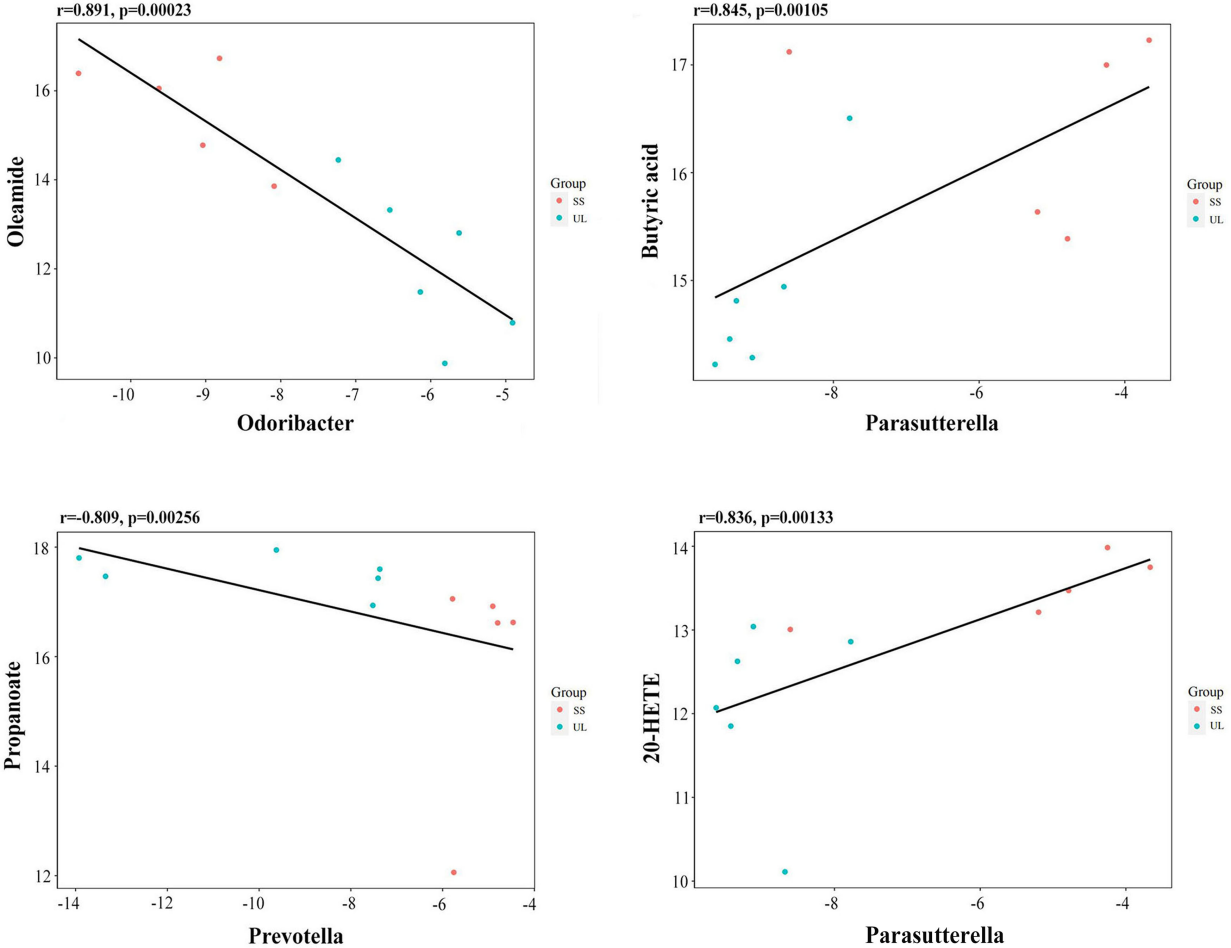
Scatter plots illustrating the statistical correlation between the relative abundance of altered gut bacterial genera and LC-MS spectrum intensities of the differential metabolites.

Spearman’s correlation coefficient was calculated to determine the functional correlations between the changes in the gut microbiome and metabolites profiles based on the difference in gut microbes and metabolites between different groups. The correlations were considered significant when r ≥ 0.8, *p-value* < 0.05. The results showed that the oleamide level was negatively correlated with *Odoribacter* abundance (r = -0.891, *p-value* = 0.00023) (**Figure 6**). The butyric acid level was positively correlated with *Parasutterella* abundance (r = 0.845, *p-value* = 0.00105) (**Figure 6**). The propanoate level was negatively correlated with *Prevotella* abundance (r = -0.809, *p-value* = 0.00256) (**Figure 6**). The 20-hydroxyeicosatetraenoic acid (20-HETE) level was positively correlated with *Parasutterella* abundance (r = 0.836, p = 0.00133) (**Figure 6**). These results suggested that the altered gut microbiome may interact with the metabolites to affect the progression ofunilateral vestibular deficit.

## Discussion

Previous studies suggested that the overlapping neuroanatomical structures between vestibular nuclei and emotion-related nuclei underlie the comorbidity of vertigo and psychiatric conditions. On the other hand, the gastrointestinal tract is inhabited by trillions of microorganisms, naturally influencing the normal physiology of the host through their collective metabolic activities. Structure and functional changes in the commensal gut microbiota are thought to be involved in the pathogenesis of many diseases (Lozupone et al., 2012). Several studies have indicated that the bidirectional communication between the brain and the gastrointestinal tract forms a complex system called the microbiota–gut–brain axis. Thus, in this study, we hypothesized that the gut microbiota may participate in the vestibular deficit comorbid with anxiety. In this context, we explored the effects of unilateral labyrinthectomy on the possible changes in the gut microbiome and metabolome.

Simon et al. described a surgical technique resulting in unilateral vestibular deficit in mice (Simon et al., 2020). After surgery, the mouse showed a fast functional compensation, which corresponded to rapid behavioral improvement during the first week postoperatively, and the completed compensation period was about 1 month (Cassel et al., 2019). Thus, to evaluate the potential role of gut microbiome in the acute stage of vestibular deficit, the samples were collected at 7 days postoperation. The results showed that the UL group possessed higher community diversity than the SS group. Moreover, altered composition of the gut microbiome in the UL group was identified, which was characterized by a high relative abundance of the *Lachnospiraceae NK4A136 group*, *Odoribacter*, and *Roseburia*, and a low relative abundance of *Prevotella* and *Parasutterella* at the genus level. *Lachnospiraceae_NK4A136_group*, *Odoribacter*, and *Roseburia* are the main producers of short-chain fatty acids (SCFAs), such as propionic, acetic, and butyric acids. *Lachnospiraceae NK4A136 group* belongs to the propionic acid-producing Lachnospiraceae family. The genera *Roseburia* and *Odoribacter* are known butyrate producers. SCFAs seem to be beneficial to health by maintaining intestinal epithelial integrity, reducing inflammation, and enhancing immune function. However, recent studies found that juvenile social isolated mice showed anxiety-like behaviors, which may be mediated by gut-derived propionic acid decreasing the expression of oxytocin receptors in the medial prefrontal cortex (Huang et al., 2021). *Roseburia* was overrepresented in healthy controls compared to patients with major depressive disorder (Zheng et al., 2016). Elevated *Roseburia* abundance was reported to be associated with alleviation of depression and anxiety-like behaviors in chronic unpredictable mild stress animal models (Qu et al., 2019). Butyrate-producing *Odoribacter* species was believed to modulate systemic inflammation through metabolite production. Moreover, the levels of circulating inflammatory markers are elevated in psychiatric patients. Therefore, it is hypothesized that a low level of colonic butyrate may increase intestinal permeability, followed by translocation of intestinal contents into systemic circulation, resulting in systemic inflammation and psychiatric conditions (Turna et al., 2020). *Prevotella* was reported to have a potential positive correlation with positive mood, and the relative abundance of *Prevotella* was higher in healthy controls (Li et al., 2016; Chung et al., 2019), which is consistent with our results. Studies on the biological function of *Parasutterella* in the host are still very limited. Cheung et al. demonstrated that the *Parasutterella* abundance was higher in patients with major depressive disorder compared to the healthy control (Cheung et al., 2019). Therefore, the role of *Parasutterella* in the vestibular disorder requires further exploration. The Tax4Fun functional prediction indicated that the abundance of tryptophan metabolism was lower in the UL group than in the SS group. Tryptophan is an essential amino acid for human protein synthesis and has been identified as a key regulator in the microbiota–gut–brain axis. Moreover, tryptophan is the only precursor of the neurotransmitter serotonin, which is well recognized to play an essential role in psychiatric disorders (Roth et al., 2021). Taken together, our results indicated that *Lachnospiraceae NK4A136 group*, *Odoribacter*, *Roseburia*, and *Prevotella* might be potential candidates for the management of vestibular deficit comorbid with anxiety.

Oleamide is an endogenous neuroactive fatty acid amide that was demonstrated to have an anxiolytic-like effect in socially isolated mice and antidepressive effects in a chronic mild stress rat model of depression (Wei et al., 2007). However, the primary action site and mechanism of oleamide remain unclear. It has been suggested that oleamide exerts its effects by increasing the levels or activity of endogenous cannabinoids (Fedorova et al., 2001). In the present study, the relative abundance of *Odoribacter* was higher in the UL group than in the SS group, while the oleamide level was negatively correlated with *Odoribacter*, which indicated that the oleamide level was lower in the UL group than in the SS group. The relative abundance of *Parasutterella* was lower in the UL group, while the butyric acid level was positively correlated with the *Parasutterella* abundance, suggesting that the butyric acid level was also lower in the UL group than in the SS group. *Prevotella* was less abundant in the UL group than in the SS group, while the propanoate level was negatively correlated with *Prevotella* abundance, which suggests that the propanoate level was higher in the UL group than in the SS group. As mentioned above, decreased butyric acid and elevated propionic acid levels contribute to higher anxiety levels. Finally, 20-HETE is one of the arachidonic acid (AA) metabolites, being the endogenous ligand for transient receptor potential cation channel subfamily V member 1 (TRPV1). By activating TRPV1, 20-HETE could aggravate neurogenic inflammation and ischemic neuronal injury (Zhang et al., 2018; Hamers et al., 2021). Moreover, TRPV1 is functionally expressed in rat vestibular ganglia (Kamakura et al., 2013). Consequently, further studies are required to elucidate the potential role of 20-HETE in the progression of vestibular deficit.

The vagus nerve is a crucial component of the microbiota–gut–brain axis and plays a crucial role in mood and anxiety disorders. Meanwhile, the gut microbiota partly influences mood and anxiety by directly affecting the vagus nerve activity (Breit et al., 2018). Balaban and Beryozkin (1994) demonstrated that vestibular nucleus projections to the dorsal motor nucleus of the vagus nerve (DMX) may contribute to the autonomic manifestations of vestibular deficit. Eren et al. (2018) demonstrated that noninvasive vagus nerve stimulation (nVNS) improved quality of life, postural balance control, depression, and anxiety in patients with persistent postural-perceptual dizziness, which is the most prevalent chronic vestibular disorder. In addition, emerging evidence suggests that nVNS ameliorate vertigo and headache as well as nystagmus in vestibular migraine patients (Beh and Friedman, 2019; Beh, 2020; Beh, 2021). This evidence suggests that the gut microbiome may participate in the comorbidity of vestibular deficit and anxiety by affecting the vagus nerve.

In summary, this study revealed that mice with vestibular deficit had significantly altered gut microbiome and metabolites profiles compared to control animals. Significantly altered microbes and metabolites are reported to participate in the pathogenesis of anxiety disorder. Therefore, this study highlighted that targeting these microbes or their metabolites could be a promising new strategy for alleviating anxiety in patients with vestibular deficit. Furthermore, the findings presented herein provide novel insights into the mechanisms underlying the comorbidity of vestibular deficit and anxiety.

There are several limitations of the present study that should be mentioned and discussed. First, this study was performed in the acute stage of vestibular deficit. It would be interesting to set more time points to observe the dynamic changes in the gut microbiome, such as by retesting in the compensation stage to evaluate the potential role of the gut microbiome in vestibular compensation. Second, the blood sample of the mouse should be tested simultaneously in a further study to verify that the differential metabolite exerts functions in the distal organ. Third, using the video-oculography test to quantify the vestibular function instead of behavioral evaluation would be more precise. Other limitations include the small sample size, the absence of clinical studies and the difficulty of translating the results from animals to humans. Finally, further investigation is needed to elucidate the specific mechanism underlying the effects of gut microbes and their metabolites on vestibular deficit-related anxiety.

## Conclusion

In conclusion, using 16S rRNA gene sequencing, we described for the first time that the gut microbiome composition of mice and gut metabolome are significantly altered after unilateral labyrinthectomy. Moreover, the differential genus and metabolites found in the gut of the mice in the UL group were in close relation to the anxiety regulation of the animals. This study represents an important step to explore further the role of the gut microbiome in the pathogenesis of vestibular deficit comorbid with anxiety. Future studies are required to demonstrate whether the gut microbiome and metabolome altered in vestibular deficit patients, as well as to investigate the role of gut microbes and metabolites in the comorbidity of vestibular deficit and anxiety from the perspective of molecular mechanisms.

## Data Availability Statement

The data presented in the study are deposited in the NCBI Sequence Read Archive repository, accession number PRJNA788014.

## Ethics Statement

The animal study was reviewed and approved by the ethics committee of the Eye, Ear, Nose, and Throat Hospital of Fudan University, Shanghai, China.

## Author Contributions

CD contributed to the conception of the study. CD and FL designed the experiment. FL, YF, and HL performed the experiments. FL, XS, HL, WL, QW, DK, and JW contributed to the analyses and interpretation. YZ helped perform the analysis with constructive discussions. FL wrote the manuscript. CD reviewed and revised the manuscript. All authors contributed to the article and approved the submitted version.

## Funding

This study was funded by the National Natural Science Foundation of China (No. 82171142, 81800910).

## Conflict of Interest

The authors declare that the research was conducted in the absence of any commercial or financial relationships that could be construed as a potential conflict of interest.

## Publisher’s Note

All claims expressed in this article are solely those of the authors and do not necessarily represent those of their affiliated organizations, or those of the publisher, the editors and the reviewers. Any product that may be evaluated in this article, or claim that may be made by its manufacturer, is not guaranteed or endorsed by the publisher.

## Supplementary Material

Supplementary Figure 1 Microbial community dynamics of the gut after sham surgery. Cladogram representation of the gut microbiota in the SS versus the CON group by 16S rRNA sequencing, n= 5 per group. Enriched taxa in the SS (green) and CON (red) groups are indicated. The brightness of each dot is correlated with its LDA effect size **(A)**. LDA coupled with effect size measurements in the SS and CON groups. Enriched taxa in the SS (green) and CON (red) groups are displayed with LDA scores. Only taxa with LDA scores beyond the threshold of 4.0 are shown **(B)**. Statistical analysis of the gut microbiota in the SS and CON groups by Student’s t-test **(C)**. CON, control group; SS, sham surgery group.

Supplementary Figure 2 Tax4Fun functional prediction of predominant taxa of gut microbiota in the UL, SS, and CON groups (UL: n = 6, CON: n = 5, SS: n = 5). The UL group showed a lower abundance of valine, leucine, and isoleucine degradation, tryptophan metabolism, and a higher abundance of NOD-like receptor signaling pathway than the SS group **(A)**. The CON group showed a higher abundance of ribosome biogenesis than the SS group **(B)**.

Supplementary Figure 3 PCA was performed to show the metabolite profile (UL: n = 6, CON: n = 5, SS: n = 5). The 2D scatter plot shows that the CON group was separated from the UL and SS groups **(A–C)**. CON, control group; UL, unilateral labyrinthectomy group; SS, sham surgery group.

Supplementary Figure 4 Volcano plot showing differential metabolites between the CON and SS groups **(A)**. Linear discriminant analysis (LDA) effect size (LEfSe) results showed the top 20 differential metabolites between the CON and SS groups **(B)**. Hierarchical cluster analysis of differential metabolites between the CON and SS groups **(C)**. Each column represents a sample and each row stands for a metabolite. The differential metabolites were identified using the following criteria: variable importance in projection (VIP) ≥1.0, *p-value* <0.05 and fold change ≥ 2 or ≤ 0.5. n= 5, per group. CON, control group; SS, sham surgery group.

Supplementary Figure 5 KEGG enrichment analysis of differential metabolites between the CON and SS groups **(A)**, and between the UL and SS groups **(B)**. UL: n = 6, CON: n = 5, SS: n = 5. KEGG, Kyoto Encyclopedia of Genes and Genomes; CON, control group; UL, unilateral labyrinthectomy group; SS, sham surgery group.
