# Supplementary figures and images for: Gut Microbiome and Metabolome Changes in Mice With Acute Vestibular Deficit
Source: Front Cell Infect Microbiol. 2022 Apr 4;12:821780. doi: 10.3389/fcimb.2022.821780 (PMC9013912; doi:10.3389/fcimb.2022.821780)

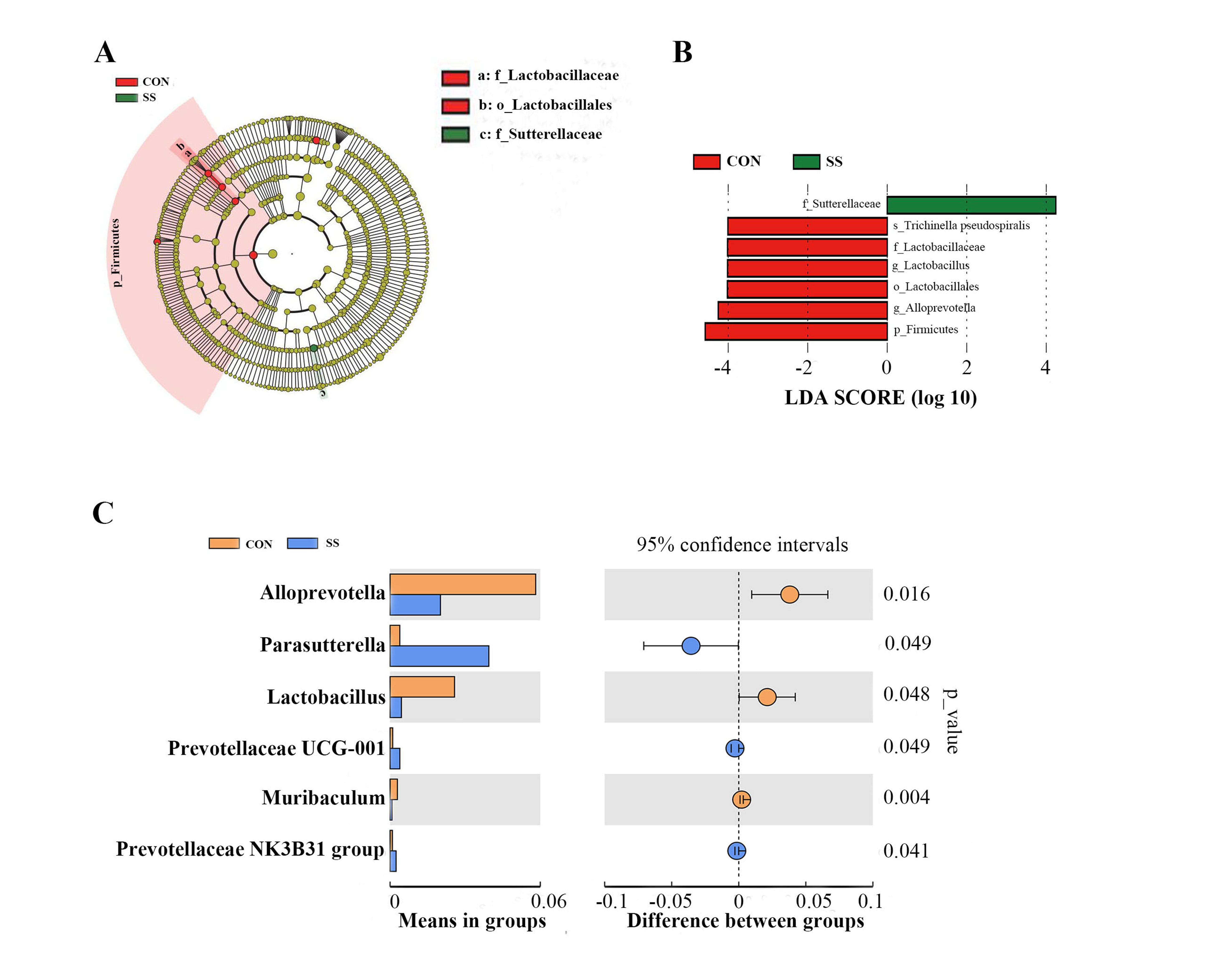

Supplement: Supplementary Figure 1 — Microbial community dynamics of the gut after sham surgery. Cladogram representation of the gut microbiota in the SS versus the CON group by 16S rRNA sequencing, n= 5 per group. Enriched taxa in the SS (green) and CON (red) groups are indicated. The brightness of each dot is correlated with its LDA effect size (A). LDA coupled with effect size measurements in the SS and CON groups. Enriched taxa in the SS (green) and CON (red) groups are displayed with LDA scores. Only taxa with LDA scores beyond the threshold of 4.0 are shown (B). Statistical analysis of the gut microbiota in the SS and CON groups by Student’s t-test (C). CON, control group; SS, sham surgery group. [file Image_1.jpeg]

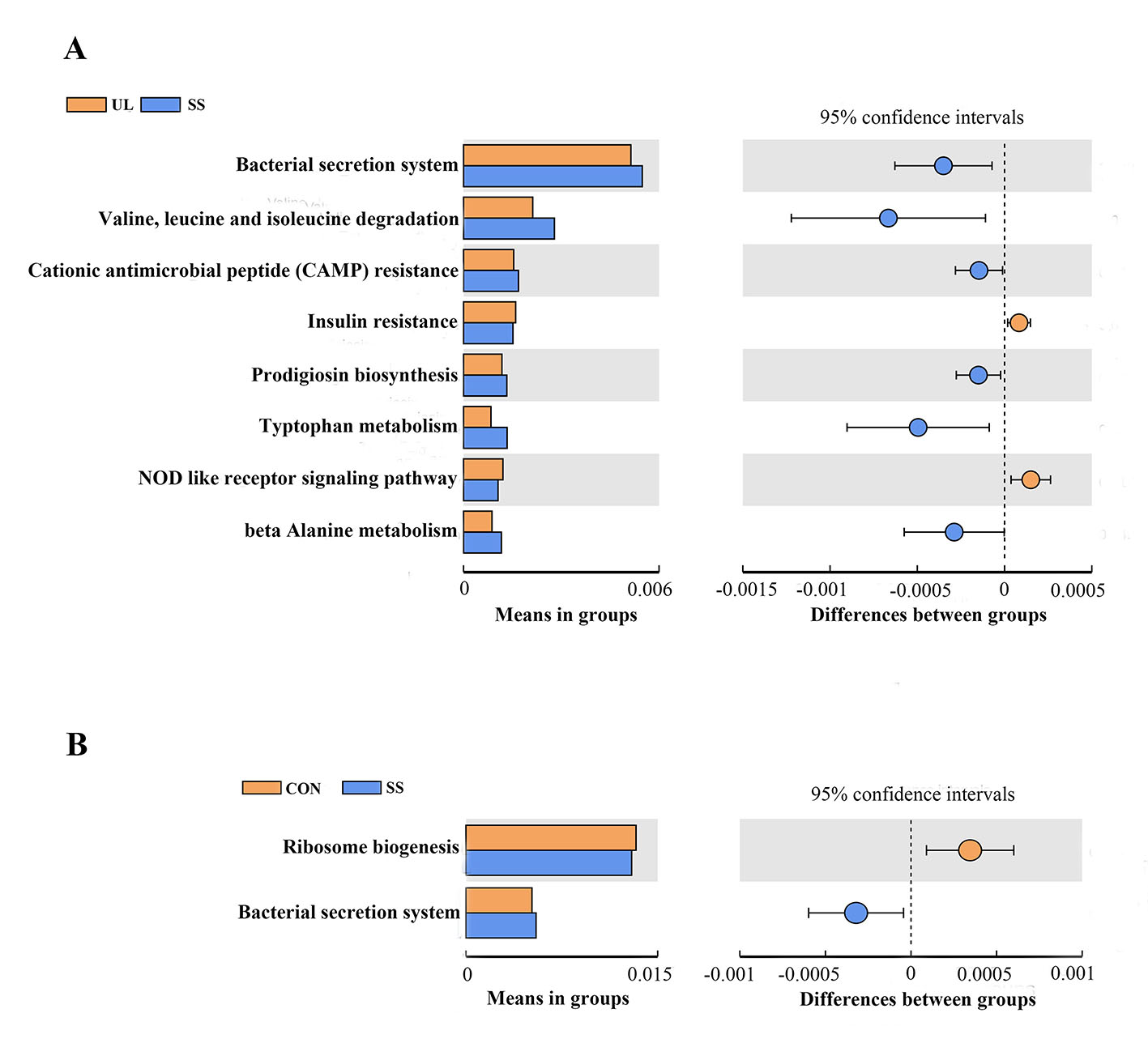

Supplement: Supplementary Figure 2 — Tax4Fun functional prediction of predominant taxa of gut microbiota in the UL, SS, and CON groups (UL: n = 6, CON: n = 5, SS: n = 5). The UL group showed a lower abundance of valine, leucine, and isoleucine degradation, tryptophan metabolism, and a higher abundance of NOD-like receptor signaling pathway than the SS group (A). The CON group showed a higher abundance of ribosome biogenesis than the SS group (B). [file Image_2.jpeg]

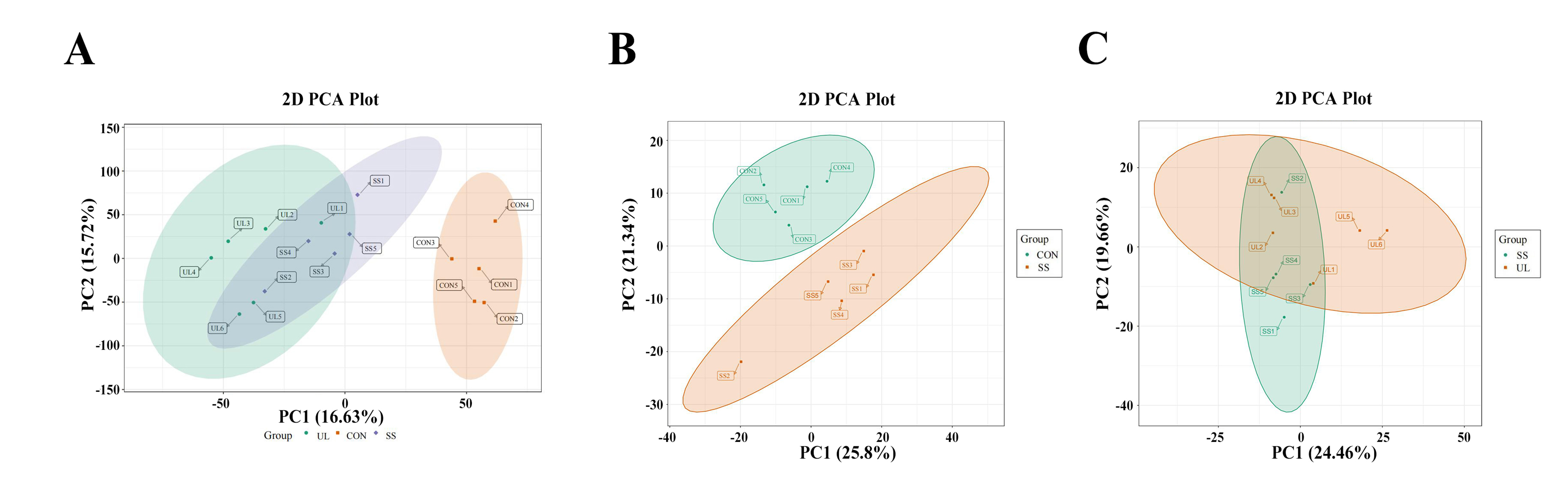

Supplement: Supplementary Figure 3 — PCA was performed to show the metabolite profile (UL: n = 6, CON: n = 5, SS: n = 5). The 2D scatter plot shows that the CON group was separated from the UL and SS groups (A–C). CON, control group; UL, unilateral labyrinthectomy group; SS, sham surgery group. [file Image_3.jpeg]

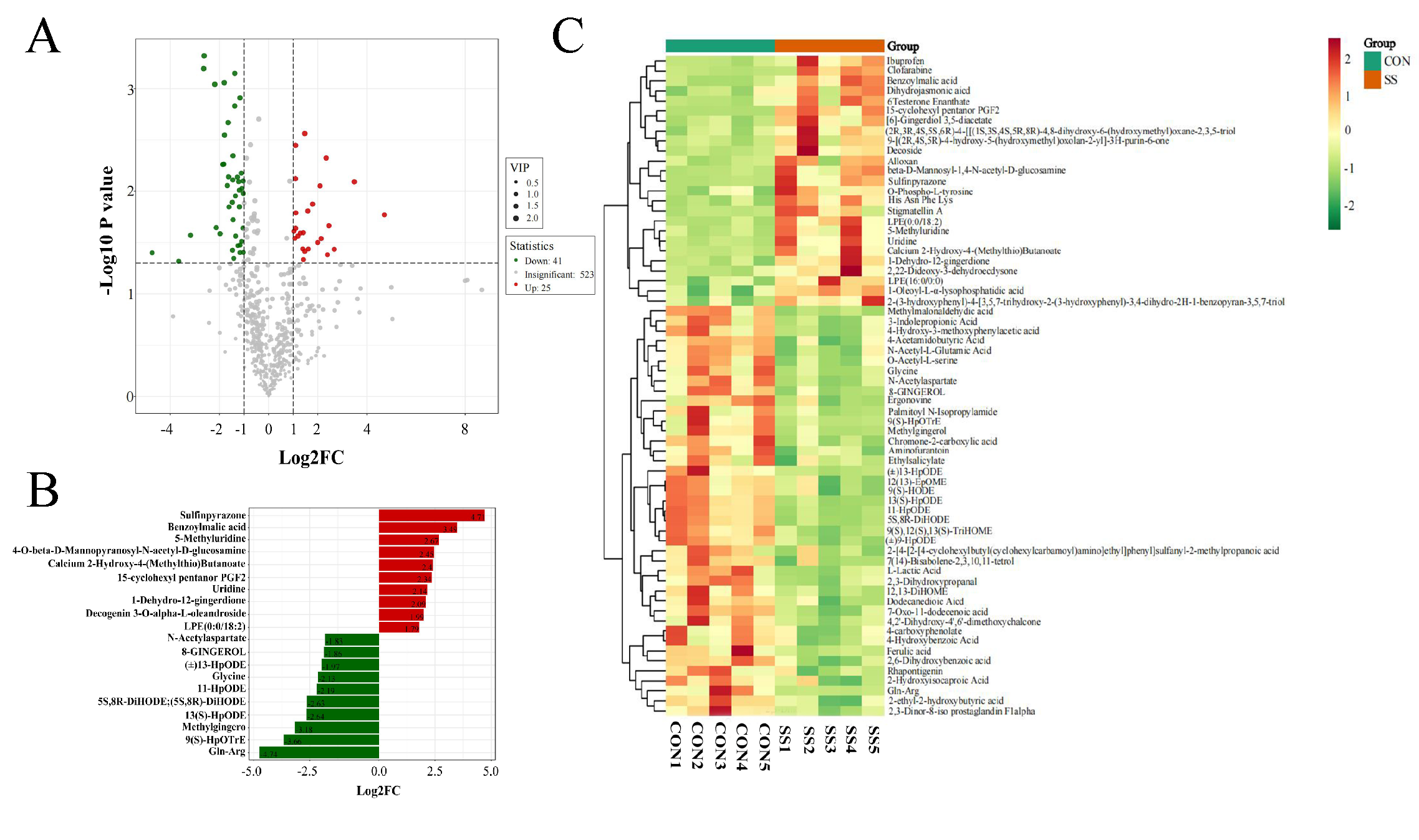

Supplement: Supplementary Figure 4 — Volcano plot showing differential metabolites between the CON and SS groups (A). Linear discriminant analysis (LDA) effect size (LEfSe) results showed the top 20 differential metabolites between the CON and SS groups (B). Hierarchical cluster analysis of differential metabolites between the CON and SS groups (C). Each column represents a sample and each row stands for a metabolite. The differential metabolites were identified using the following criteria: variable importance in projection (VIP) ≥1.0, p-value <0.05 and fold change ≥ 2 or ≤ 0.5. n= 5, per group. CON, control group; SS, sham surgery group. [file Image_4.tif]

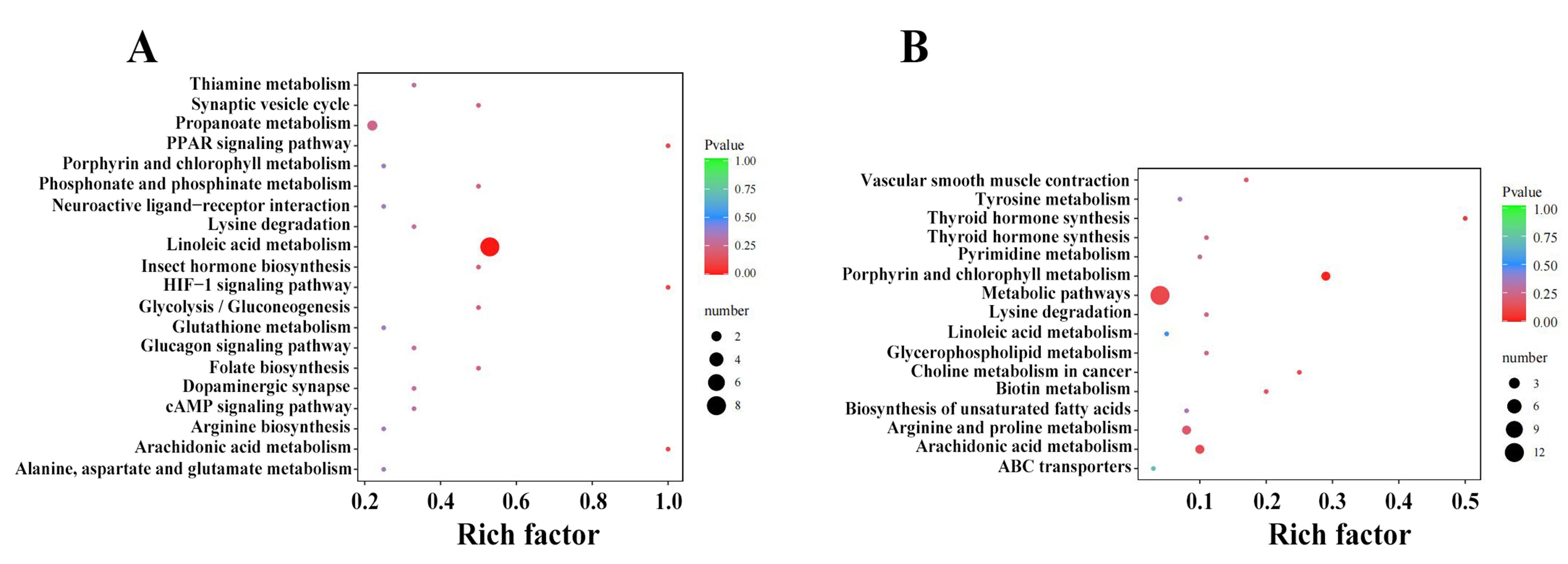

Supplement: Supplementary Figure 5 — KEGG enrichment analysis of differential metabolites between the CON and SS groups (A), and between the UL and SS groups (B). UL: n = 6, CON: n = 5, SS: n = 5. KEGG, Kyoto Encyclopedia of Genes and Genomes; CON, control group; UL, unilateral labyrinthectomy group; SS, sham surgery group. [file Image_5.jpeg]
